# Analysis of Lung Microbiome in COVID-19 Patients during Time of Hospitalization

**DOI:** 10.3390/pathogens12070944

**Published:** 2023-07-17

**Authors:** Linlin Xie, Liangjun Chen, Xinran Li, Junying Zhou, Hongpan Tian, Jin Zhao, Zhiqiang Li, Yirong Li

**Affiliations:** 1 Department of Laboratory Medicine, Zhongnan Hospital of Wuhan University, Wuhan 430071, China; xielinlin@whu.edu.cn (L.X.); chenliangjun1122@163.com (L.C.);; 2 Department of Neurosurgery, Zhongnan Hospital of Wuhan University, Wuhan 430071, China; 3 Wuhan Research Center for Infectious Diseases and Tumors, Chinese Academy of Medical Sciences, Wuhan 430071, China; 4 Hubei Engineering Center for Infectious Disease Prevention, Control and Treatment, Wuhan 430071, China

**Keywords:** COVID-19, SARS-CoV-2, lung microbiota, nanopore sequencing

## Abstract

Background: Severe acute respiratory syndrome coronavirus 2 (SARS-CoV-2), which is the pathogenic agent of the rapidly spreading pneumonia called coronavirus disease 2019 (COVID-19), primarily infects the respiratory and digestive tract. Several studies have indicated the alterations of the bacterial microbiome in the lower respiratory tract during viral infection. However, both bacterial and fungal microbiota in the lung of COVID-19 patients remained to be explored. Methods: In this study, we conducted nanopore sequencing analyses of the lower respiratory tract samples from 38 COVID-19 patients and 26 non-COVID-19 pneumonia controls. Both bacterial and fungal microbiome diversities and microbiota abundances in the lung were compared. Results: Our results revealed significant differences in lung microbiome between COVID-19 patients and non-COVID-19 controls, which were strongly associated with SARS-CoV-2 infection and clinical status. COVID-19 patients exhibited a notably higher abundance of opportunistic pathogens, particularly *Acinetobacter baumannii* and *Candida* spp. Furthermore, the potential pathogens enriched in COVID-19 patients were positively correlated with inflammation indicators. Conclusions: Our study highlights the differences in lung microbiome diversity and composition between COVID-19 patients and non-COVID-19 patients. This may contribute to predicting co-pathogens and selecting optimal treatments for respiratory infections caused by SARS-CoV-2.

## 1. Introduction

The severe acute respiratory syndrome coronavirus 2 (SARS-CoV-2) causes a severe pneumonia known as coronavirus disease 2019 (COVID-19), as defined by the World Health Organization (WHO) [1,2]. Since the outbreak in December 2019, SARS-CoV-2 has spread globally, taking millions of lives worldwide. Due to its high mortality rate and global threat [3], intensive research has investigated the body’s response against this virus, potential co-infections or secondary infections, and the optimal therapies for patients [4,5,6,7].The human respiratory tract is the major portal of entry for SARS-CoV-2 and site of serious clinical manifestations and contains an airway microbiome representing its microenvironment and serving as an essential component of the airway epithelial barrier. The respiratory microbiota interacts with the host immune system, thereby influencing clinical outcomes in chronic and acute respiratory diseases [8,9]. During viral respiratory infections such as the influenza virus, alterations in the respiratory microbiota can potentially modify immune response, leading to co-infections and secondary infections [10,11,12]. For example, disruption of the respiratory microbiome can affect the host’s innate immune response during influenza A virus infection [13]. Simultaneously, the respiratory microbiome can also influence the colonization and proliferation of pathogens [14,15]. Increasing evidence has indicated that SARS-CoV-2 infection could potentially modify the nasal, oropharyngeal, and lung microenvironment, causing microbial dysbiosis [16,17,18,19,20,21,22]. This dysbiosis could lead to a co-infection or secondary infection, resulting in higher morbidity and mortality of COVID-19 patients [23,24,25,26,27]. Therefore, understanding the association between the respiratory microbiota and COVID-19 pathogenesis provides an important foundation for formulating better diagnostic and management strategies for combating the pandemic. Due to limitations in sampling techniques, investigations into the microbiome in the lung have been restricted, as the lower respiratory tract is less accessible. Previous small-scale studies have focused on describing the bacterial composition in the lungs during COVID-19 [17,22,28]. However, the identification of other microorganisms, such as fungi, which may also impact lung health, has been neglected [29,30]. There is a lack of research exploring both bacterial and fungal microbiota in the lungs of COVID-19 patients. Further studies on the lung microbiota are addressed to further understand the bacterial and fungal composition in the long after the SARS-CoV-2 infection, and to distinguish the differences in the lung microbiota of COVID-19 patients compared to other pneumonia patients. In this study, we used nanopore sequencing to analyze bacterial and fungal microbiota in the lung samples from 38 COVID-19 patients and 26 non-COVID-19 pneumonia controls. Our results revealed significant differences in the lung microbiome between COVID-19 patients and non-COVID-19 controls. COVID-19 patients exhibited higher abundances of opportunistic pathogens, particularly *Acinetobacter baumannii* and *Candida* spp. We examined potential confounders and found that SARS-CoV-2 infection and clinical status explained most variation in the lung microbiome. Furthermore, potential pathogens enriched in COVID-19 patients correlated with changes in some inflammation indicators.

## 2. Materials and Methods

### 2.1. Patients and Sample Collection

Sputum and bronchoalveolar lavage fluid (BALF) samples were collected from 38 hospitalized pneumonia patients with laboratory-confirmed SARS-CoV-2 infection (COVID-19) and 26 non-COVID-19 pneumonia patients at Zhongnan Hospital of Wuhan University, Wuhan, China. COVID-19 was confirmed by at least 2 times of RT-PCR tests, while non-COVID-19 patients tested negative for SARS-CoV-2. Clinical and laboratory data were obtained from the electronic medical record. Samples were collected on 1 February to 15 August 2020.This study was approved by the Ethics Committee of Zhongnan Hospital (No. 2021024), Wuhan University. All patients signed an informed consent.

### 2.2. Microbial DNA Extraction

Microbial DNA was extracted within 4 h after collection samples using the Sansure DNA Extraction Kit (Changsha, China) according to the manufacturer’s instruction. The extracted DNA was dissolved in double distilled water and stored at −80 °C before further analysis.

### 2.3. Nanopore-Targeted Sequencing and Analysis

Nanopore-targeted sequencing contains the following three steps, amplification, library construction, and sequencing.

#### 2.3.1. Amplification

Microbial 16s rRNA gene and fungal internal transcribed spacers 1 and 2 (ITS1/2) were amplified by two respective PCR assays using universal primers. For bacteria, 16s rRNA genes were amplified using primers 27F (5′-AGRGTTYGATYMTGGCTCAG-3′) and 1492R (5′-RGYTACCTTGTTACGACTT-3′) [31]. For fungi, the ITS1/2 regions were amplified using primers ITS1 (5′-TCCGTAGGTGAACCTGCGG-3′) and ITS4 (5′-TCCTCCGCTTATTGATATGC-3′) [32]. Amplification of the 16s rRNA gene was carried out in a 20 μL reaction system, including 8 μL extracted DNA, 2 μL barcoded primer (10 μM), and 10 μL 2× KOD OneTM PCR Master Mix (TOYOBO) using the following procedure: 1 cycle at 98 °C for 3 min and 35 cycles at 98 °C for 10 s, 55 °C for 5 s, and 68 °C for 10 s, followed by a final elongation step at 68 °C for 5 min. Fungal ITS1/2 were first amplified using the same reaction system and PCR conditions using the primer mix without barcode, then the PCR products were purified with 0.8× AMpure beads (Beckman Coulter) and eluted in 10 μL Tris-EDTA (TE) buffer. Subsequently, 5 μL eluate was used as template for PCR with 5 μL barcoded ITS1/2 primer set (10 μM) and 10 μL 2× Phusion U Multiplex PCR Master Mix using the following procedure: 1 cycle at 98 °C for 3 min and 10 cycles at 98 °C for 10 s, 55 °C for 5 s, and 68 °C for 5 s, followed by a final elongation step at 68 °C for 5 min. The barcoded products of the 16s rRNA gene and ITS1/2 amplification from the same samples were pooled according to a mass ratio of 10:3. Two negative controls, including amplified negative control and extracted negative control, were prepared for library construction and meta-transcriptomic sequencing in parallel with the clinical samples in each batch of our experiments.

#### 2.3.2. Library Construction

The pooled products from the different samples were mixed equally and used to construct sequencing libraries using the 1D Ligation Kit (SQK-LSK109; Oxford Nanopore, Oxford, UK).

#### 2.3.3. Sequencing and Analyzing

The library was sequenced on Oxford Nanopore MinION. TE buffer was assayed in each batch as a negative control. Raw Fast5 files were basecalled and demultiplexed in real-time using Albacore (v2.3.1). Reads of less than 7 nucleotide acids were filtered out. Porechop (v0.2.4) was also used to trim the barcodes and the adapters from raw reads. Then, the filtered sequencing reads were mapped to reference databases downloaded from 16S rDNA/ITS reference database collected from NCBI FTP (ftp://ftp.ncbi.nlm.nih.gov/refseq/TargetedLoci) accessed on 1 October 2020 using Blast with an E-value cutoff of 1 × 10^−5^. Taxonomy of each read was assigned according to the taxonomic information of the mapped subject reference with the highest identity and coverage. A consensus sequence was generated for reads assigned to the same species Medaka (v.0.10.1) and re-mapped to the reference database. The species-level taxonomy of the mapped subject reference was detected as the final assignment. The positive bacteria or fungi were determined if they met any of the following thresholds, as described previously [33]. Bacteria out of the critical list: mapped reads of microbes (species level) in the sample >100 or mapped reads of microbes (species level) in the sample is greater than that of any other microbes, and the ratio of mapped reads in the sample and negative control >10. Fungi out of the critical list: >20 reads at the species level, or the relative abundance is higher than 50%. The ratio of mapped reads in the sample and negative control >10.

### 2.4. Statistical Analysis

All analyses were performed in R statistical framework. Patient characteristics were compared between groups using chi-square tests for categorical variables and independent *t*-tests for continuous variables. Microbial diversity was estimated using the phyloseq and vegan packages implemented in R. Alpha diversity was compared between groups with the Wilcoxon rank-sum test. Beta diversity was calculated as Bray Curtis dissimilarity and compared between groups using permutational multivariate analysis of variance (PERMANOVA). Linear discriminant analysis (LDA) effect size (LEfSE) on the Galaxy platform identified differentially abundant genera. DESeq2 analysis was used to identify differentially abundant species. To explore potential associations between the lung species-level microbiota composition and the extensive metadata, distance-based redundancy analyses (dbRDA) were performed using the capscale function from vegan in univariate models. Model *p* values were corrected using Benjamini-Hochberg’s (BH) multiple-testing correction to select significant variables with BH-adjusted *p* values < 0.05. These significant variables were included in a multivariate model, and nonredundant contribution to variation was calculated using forward stepwise variable selection via the ordiR2step function from vegan. Correlations between clinical indicators and the lung microbiota were analyzed using the Spearman Correlation Analysis test. All statistical analyses were performed using packages under R version 4.1.1. A *p*-value of less than 0.05 was regarded as statistically significant.

## 3. Results

### 3.1. Clinical Characteristics of the Study Population

This study included 38 hospitalized COVID-19 patients and 26 non-COVID-19 patients (Supplementary Table S1). Clinical characteristics, including hematological, medical, and biochemical results, are demonstrated in Table 1. The mean age and gender ratio were comparable between COVID-19 and non-COVID-19 patients (*p* > 0.05). Over 75% of patients in both groups had comorbidities, including diabetes, hypertension, coronary artery disease, chronic lung disease, and chronic infectious disease. COVID-19 patients required significantly more oxygen support (*p* = 0.001), including noninvasive ventilation, invasive ventilation, and ECMO, compared to non-COVID-19 patients. All patients received antibiotics treatment, while antiviral and antifungal treatment were significantly more frequent in COVID-19 patients (*p* < 0.01). Among these patients, 12 COVID-19 patients and 6 non-COVID-19 patients died during hospitalization, respectively. Laboratory-tested indicators showed that COVID-19 patients had significantly decreased lymphocyte counts (*p* = 0.002) and hemoglobin levels (*p* = 0.015) compared to non-COVID-19 patients, which is consistent with previous reports [34]. In contrast, COVID-19 patients showed increased neutrophil to white blood cell ratio (*p* = 0.001) and serum amyloid A (SAA) level (*p* = 0.048). These key laboratory differences may reflect the distinct immunopathogenesis of COVID-19 compared to other pneumonia etiologies.

### 3.2. Lung Microbiome Diversity in COVID-19 and Non-COVID-19 Patients

**Figure 1:**
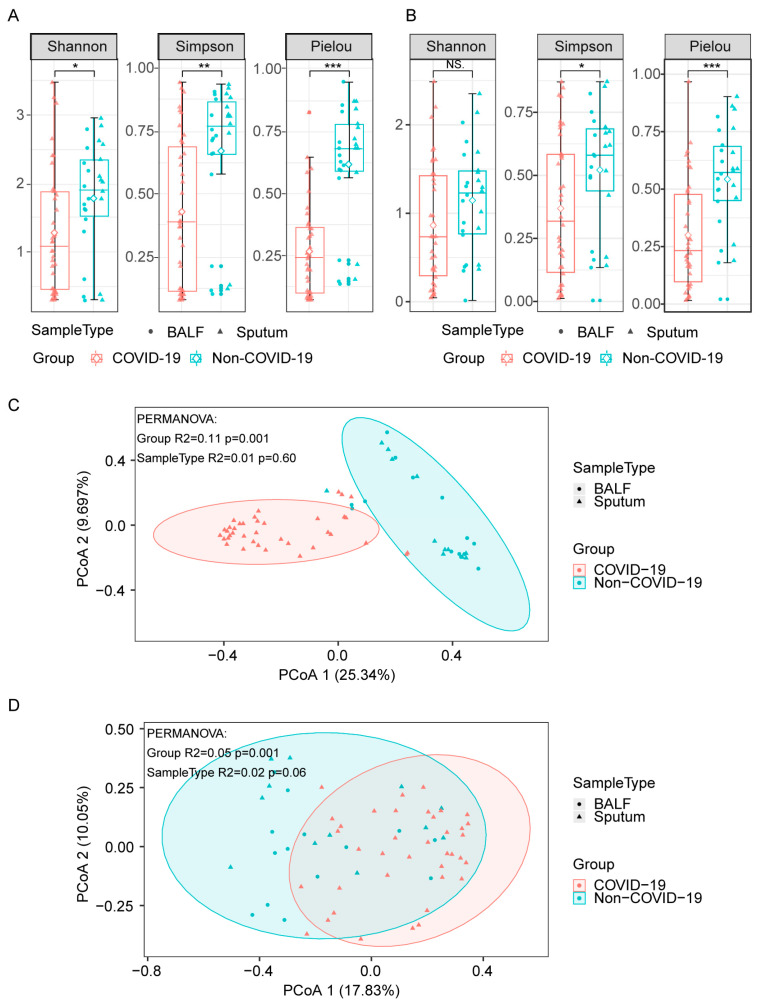
Diversities of lung microbiota in COVID-19 and non-COVID-19 patients. (**A**,**B**), α-diversity indices of bacteria (**A**) and fungi (**B**) in COVID-19 and non-COVID-19 patients. Differences between groups were analyzed using the Wilcoxon rank-sum test (*** *p* ≤ 0.001, ** *p* ≤ 0.01, * *p* ≤ 0.05, NS., *p* > 0.05). (**C**,**D**), PCoA (Principal Coordinates Analysis) based on Bray–Curtis dissimilarities for the bacterial (**C**) and fungal (**D**) β-diversity in COVID-19 and non-COVID-19. Group differences were tested by pairwise permutational multivariate analysis of variation (PERMANOVA).

To investigate the profiling of lung microbiota, we collected sputum or BALF samples from patients and sequenced them using a rapid nanopore sequencing technique (Supplementary Table S2). We determined the bacterial and fungal species present in the samples, as well as their corresponding abundances. To explore the biodiversity in lung microbiota, we utilized the Shannon, Simpson, and Pielou diversity index for the α-diversity analysis, and principal coordinate analysis (PCoA) for the β-diversity analysis. As shown in Figure 1, the α-diversity indices of bacteria (Figure 1A) and fungi (Figure 1B) were significantly lower in COVID-19 patients compared to non-COVID-19 patients, indicating a decreased biodiversity in COVID-19 patients. In addition, PcoA β-diversity analysis showed distinct bacterial compositions between the two groups (Figure 1C, R^2^ = 0.13, *p* = 0.001, with PERMANOVA test), while the differences in fungal compositions were less pronounced in non-COVID-19 and COVID-19 patients (Figure 1D, R^2^ = 0.05, *p* = 0.001, with PERMANOVA test).

### 3.3. Lung Microbiome Composition in COVID-19 and Non-COVID-19 Patients

**Figure 2:**
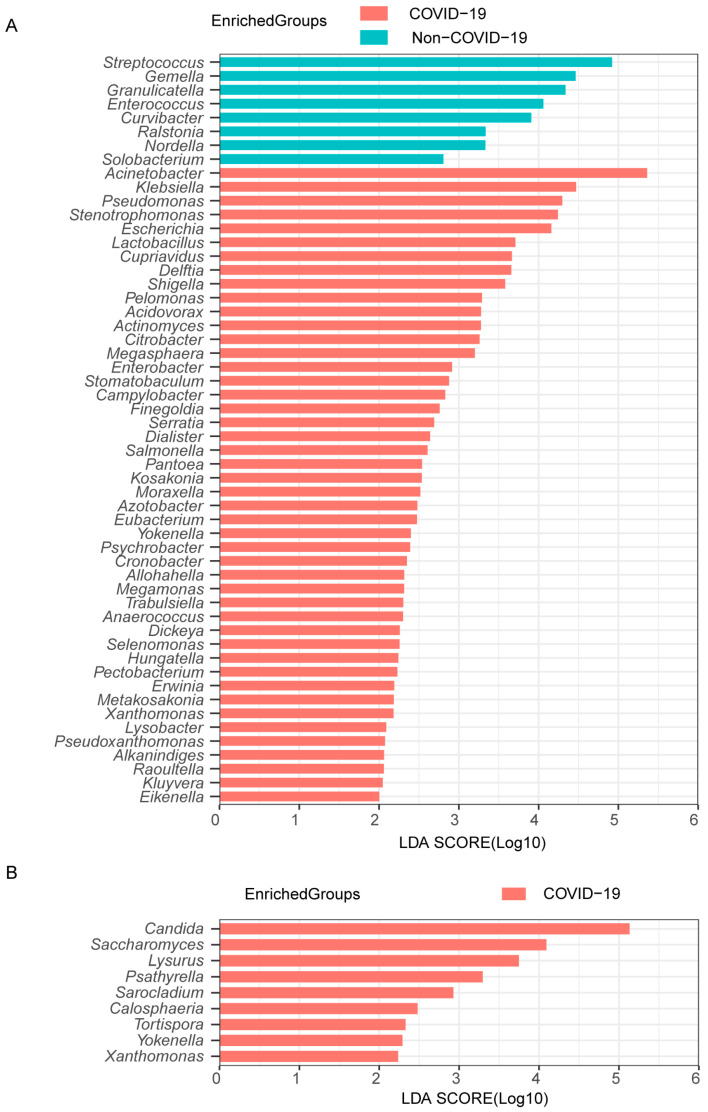
Differentially abundant genera in COVID-19 and non-COVID-19 patients. The bacterial (**A**) and fungal (**B**) taxa with significant differences between the COVID-19 and non-COVID-19 patients were identified by LDA effect size (LEfSe) analysis. Linear discrimination analysis (LDA)-score threshold > 2.

To further analyze the lung microbiota community in more detail, we examined the changes in each microbiome genus between non-COVID-19 and COVID-19 patients. COVID-19 patients had a predominance of *Acinetobacter*, *Klebsiella,* and *Pseudomonas* in their bacterial microbiome, whereas non-COVID-19 patients had a bacterial composition characterized by *Streptococcus*, *Staphylococcus,* and *Gemella* (Supplementary Figure S1A). COVID-19 patients had dominant fungal communities consisting of *Candida* and *Yarrowia*, while *Candida*, *Yarrowia,* and *Aspergillus* were frequently observed in non-COVID-19 patients (Supplementary Figure S1B). The LefSe analysis identified 46 differentially abundant bacterial genera enriched in COVID-19 patients, predominantly opportunistic pathogens like *Acinetobacter, Klebsiella, Pseudomonas, Stenotrophomonas,* and *Escherichia*. In the case of non-COVID-19 patients, 8 types of bacteria were identified as the most prevalent, including *Streptococcus, Gemella,* and *Granulicatella* (Figure 2A). For fungi, 9 genera were differentially abundant specifically in the COVID-19 group, especially *Candida* and *Saccharomyces* (Figure 2B). Overall, COVID-19 demonstrates a highly disturbed lung microbiota composition compared to other pneumonias.

**Figure 3:**
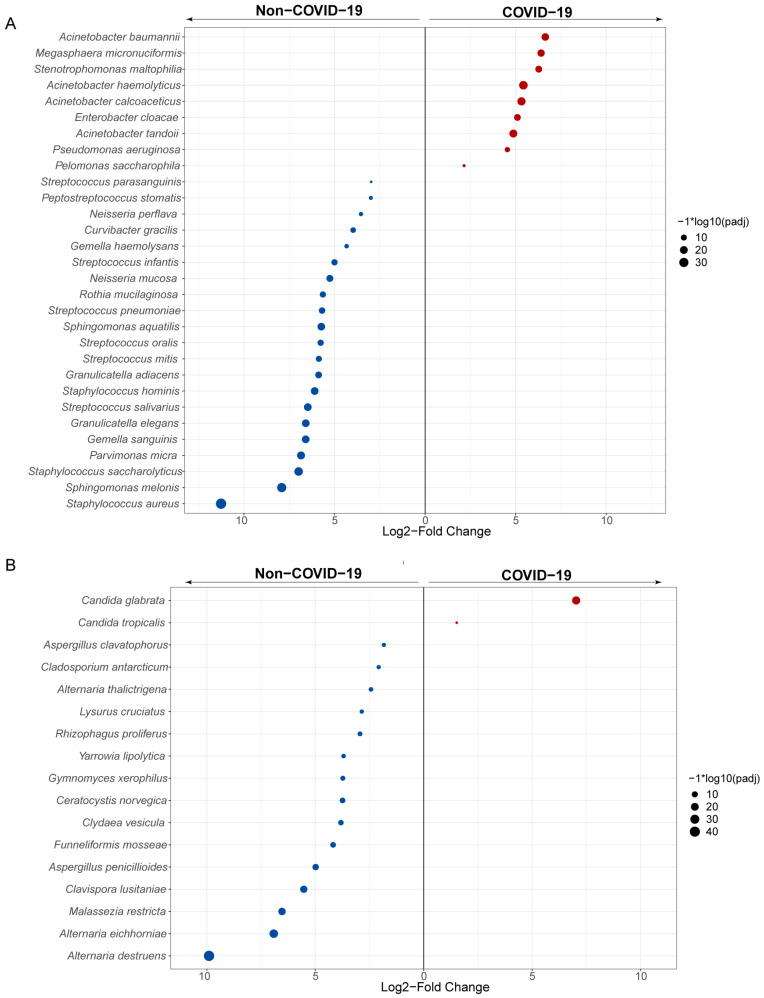
Differentially abundant species in COVID-19 and non-COVID-19 patients. Panels show taxa classified at bacterial (**A**) and fungal (**B**) species, which are differentially enriched (DESeq2, Benjamini-Hochberg Padj < 0.05, Log2FC ≥ 1.5) across the comparison between COVID-19 (red) and non-COVID-19 (blue) patients. Each point represents one taxon, scaled in size denoting −log10 (adjusted *p*-value).

Furthermore, we conducted differential expression analysis of bacterial and fungal species between COVID-19 and non-COVID-19 patients using DESeq2 (with adjusted *p* < 0.05). Multiple taxa showed differential abundant between COVID-19 and non-COVID-19 patients, with alterations observed in numerous species within the genera *Acinetobacter*, *Streptococcus*, *Neisseria*, *Staphylococcus*, *Stenotrophomonas,* and *Candida* (Figure 3A,B). The opportunistic pathogens *Acinetobacter baumannii*, *Stenotrophomonas maltophilia* and *Candida glabrata* (*Nakaseomyces glabrata)* were highly enriched in COVID-19. In contrast, bacteria associated with healthy respiratory microbiota, like *Staphylococcus aureus* and *Streptococcus salivarius*, and fungi like *Alternaria destruens* were decreased in COVID-19 patients. The enrichment of specific pathogens provides evidence for dysbiosis of the lung microbiome associated with COVID-19, which may impact disease severity. 

### 3.4. Codetection of Pathogens in the Lung of COVID-19 Patients

**Figure 4:**
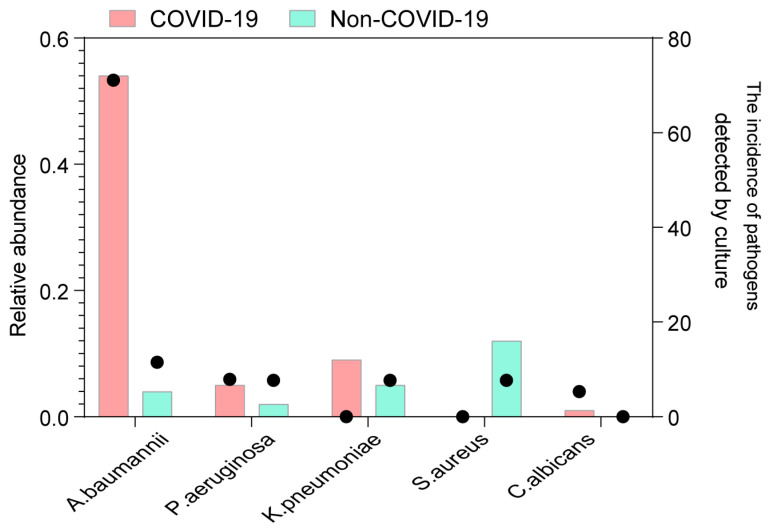
Codetection of pathogens in COVID-19 and non-COVID-19 patients. The incidence of potential pathogens in different groups. The circle shows the incidence of each pathogen by clinical microbiological culture (right *y*-axis), and the bar plot shows the median abundance of the pathogen in positive samples (left *y*-axis). The potential pathogens enriched in patients with COVID-19 are labeled in bold (Fisher’s exact test).

Subsequently, all samples were screened for common respiratory pathogens using clinical microbiological culture (Supplementary Table S3). A total of 32 COVID-19 patients had at least one pathogen besides SARS-CoV-2, and the proportion of patients with detected pathogens was significantly higher in COVID-19 patients than in non-COVID-19 patients (84.2% vs. 30.8%, *p* = 0.001). Specifically, *Acinetobacter baumannii* and *Candida albicans* were more prevalent in COVID-19 patients than in non-COVID-19 patients (Figure 4). *Acinetobacter baumannii* was identified in only 3 out of 26 non-COVID-19 patients, whereas a significantly higher proportion of COVID-19 patients (27 out of 38) were positive for *Acinetobacter baumannii* (*p* = 0.001). Moreover, the relative abundance of *Acinetobacter baumannii* was substantially higher in COVID-19 patients (*p* < 0.001). Among the identified fungi, *Candida albicans*, an opportunistic yeast, exhibited higher abundances and was exclusively detected through clinical culture in COVID-19 patients (2/38). Notably, the abundances of these two species were significantly higher in COVID-19 patients, indicating co-infection or secondary infection during hospitalization.

### 3.5. Associations between Lung Microbiome Composition and Clinical Practices in COVID-19 and Non-COVID-19 Patients

**Figure 5:**
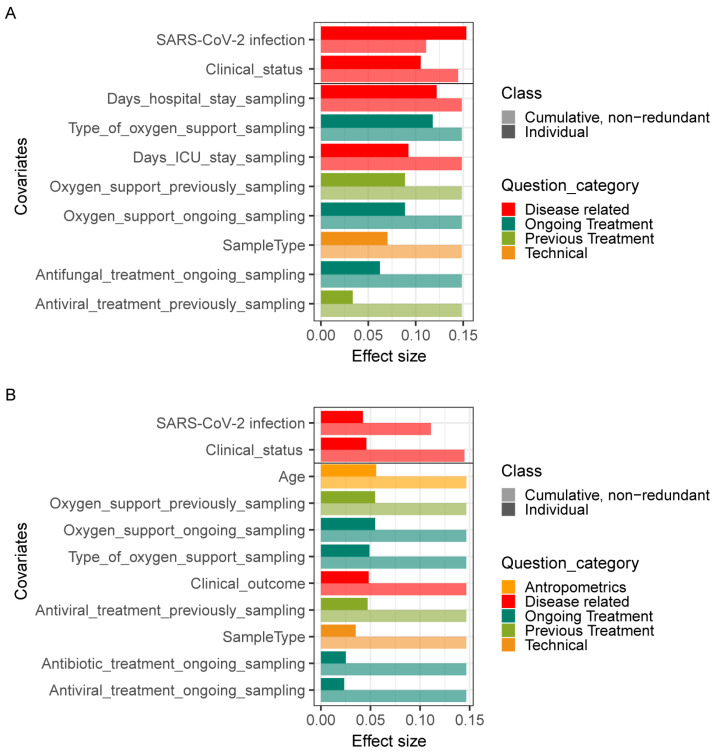
Effects of covariates on lung microbiome. Significant (BH-corrected *p* value < 0.05) covariates explaining bacterial (**A**) and fungal (**B**) variation in the lung are identified by dbRDA analysis. Individual covariates are listed on the *y*-axis; their color corresponds to the metadata category they belong to. Darker colors refer to the individual variance explained by each of these covariates assuming independence, while lighter colors represent the cumulative and nonredundant variance explained by incorporating each variable into a model using a stepwise dbRDA analysis. The black horizontal line separates those variables that are significant in the nonredundant analysis on top from the rest.

We then explored potential associations between the species-level microbiota composition in the lungs and the clinical practices collected in this study. In total, 17 covariates related to patient anthropometrics, clinical status, and treatment at sample collection were tested, including age, gender, oxygen support, and clinical medication. Individually, 10 of these covariates demonstrated a significant correlation with bacterial composition in univariate analysis (dbRDA, FDR < 0.05). These significant covariates were related to disease and clinical treatment, such as SARS-CoV-2 infection, clinical status, the number of days in hospital/ICU, and the type of oxygen support at the time of sampling (Figure 5A). In terms of fungal composition, 12 covariates exhibited a significant effect in the univariate analysis, including SARS-CoV-2 infection, clinical status, clinical outcome, the number of days in hospital, and age (Figure 5B). Notably, of these significant covariates, only two accounted for nonredundant variation of both bacteria and fungi in this dataset in a multivariate analysis (dbRDA, *p* = 0.002). These were the SARS-CoV-2 infection and clinical status at the time of sampling. These results further show that SARS-CoV-2 infection changed both bacterial and fungal composition in patients.

### 3.6. Correlation between the Lung Microbiota and Clinical Indicators during Time of Hospitalization

**Figure 6:**
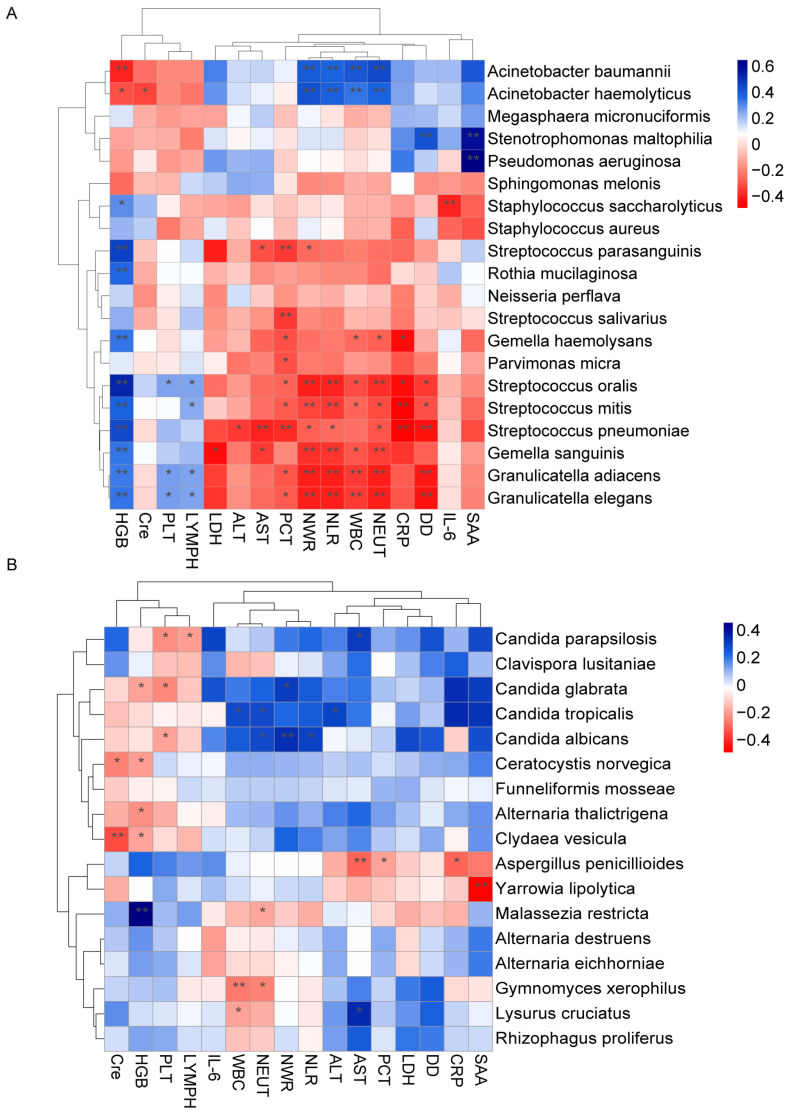
Correlation between the lung microbiota and clinical indicators. Heatmap showing the partial Spearman’s correlation coefficients between clinical indicators and differentially abundant bacterial (**A**) and fungal (**B**) species. The blue color represents a positive correlation, and the red color represents a negative correlation (** *p* ≤ 0.01, * *p* ≤ 0.05).

We further investigated the association between differentially abundant lung microbiota and clinical indicators. It revealed an association between the bacterial taxa enriched in COVID-19 and the elevated levels of multiple inflammation indicators, such as serum amyloid protein (SAA), interleukin-6 (IL-6), D-dimer (DD), procalcitonin (PCT), neutrophil counts (NEUT), leukocyte counts (WBC), neutrophil to lymphocyte ratio (NLR), and neutrophil to white blood cell ratio (NWR) (Figure 6A). For example, *Stenotrophomonas maltophilia* and *Pseudomonas aeruginosa* showed significantly strong positive correlations with SAA, while *Acinetobacter baumannii* and *Acinetobacter haemolyticus* were positively correlated with NEUT, WBC, NLR, and NWR. Strikingly, the opposite trend was observed between these inflammation indicators and the taxa with decreased abundance in COVID-19, including several species of *Streptococcus*. However, hemoglobin (HGB) levels, blood platelet counts (PLT), and lymphocyte counts (LYMPH) showed a positive correlation with taxa that had decreased abundance in COVID-19. Conversely, fewer significant associations were observed between these fungal taxa and clinical indicators (Figure 6B). *Candida parapsilosis, Candida albicans,* and *Candida glabrata* (*Nakaseomyces glabrata)*, which were enriched in COVID-19, showed a significantly negative correlation with PLT. In summary, these findings indicate that changes in the lung microbiome, particularly bacterial taxa, could influence disease severity and progression.

## 4. Discussion

Infection with the SARS-CoV-2 virus can be severe to lethal in certain populations, such as people with elder age, underlying medical conditions, and weakened immune systems. The host responses and alterations in the microenvironment upon SARS-CoV-2 invasion can determine the outcomes of patients. Several studies have indicated changes in the lung microbiota in patients with virus infection during COVID-19 [17,22,28]. However, most of these studies focused primarily on the bacterial microbiome, there is a general lack of comprehensive investigation into the entire bacterial and fungal microbial population following SARS-CoV-2 infection. An early study has found that the most prevalent bacterial and fungal genera in lung biopsies of 20 deceased COVID-19 patients were *Acinetobacter* (80.70%) and *Cutaneotrichosporon* (28.14%), respectively [35]. Our study provides a comprehensive analysis of bacterial and fungal alterations in COVID-19. We found COVID-19 patients had significantly decreased microbial diversity and more severe microbial dysbiosis compared to non-COVID-19 pneumonia patients. Both bacterial and fungal distribution in COVID-19 patients significantly differed from those in non-COVID-19 patients, as they exhibited enriched opportunistic pathogens. The predominance of these pathogens suggests SARS-CoV-2 infection disrupted the lung ecology, which may promote coinfections or secondary infections and impact disease severity. Elucidating the microbiome-virus interactions in COVID-19 is warranted to understand mechanisms and develop microbiome-targeted therapies to improve outcomes.

Consistent with previous cohort studies, co-infection with other opportunistic pathogens is the common feature of SARS-CoV-2 infection. Among the bacteria identified in our study, *Acinetobacter* was the predominant pathogen found in a significantly high abundance in majority of COVID-19 patients. *Acinetobacter*, an opportunistic pathogen, is commonly observed in nosocomial infections, particularly in intensive care units [36,37]. Importantly, patients infected with *Acinetobacter* experienced higher disease severity and mortality rates [38]. Studies have shown that 8.4–36.5% of patients with hospital-acquired *Acinetobacter* infection end up dead [39,40]. Notably, 8 out of 12 deceased COVID-19 patients had *Acinetobacter baumannii* co-infection by clinical culture. Metagenomic sequencing also revealed drastic increases in *Acinetobacter baumannii* during hospitalization compared to controls. The predominance of this deadly pathogen suggests disruption of lung ecology by SARS-CoV-2 may increase susceptibility to lethal secondary infections. Our results align with previous studies indicating an outsized role for *Acinetobacter* co-infections in severe and fatal COVID-19 cases [35,41,42]. Further research should delineate mechanisms of *Acinetobacter* overgrowth in the COVID-19 lung and develop treatments targeting this bacteria-virus interaction to potentially improve outcomes.

The fungal burden is typically low in healthy individuals, but more stable fungal communities can colonize the lung when its physiology is altered [43]. Although there is limited research on the lung mycobiome, increasing evidence suggests that the fungal microbiota is altered in COVID-19 patients [27]. A recent study reported an association between SARS-CoV-2 infection and lung dysbiosis, characterized by a shift towards *Candida* species colonization and a decrease in fungal diversity [44]. In our study, we also observed an enrichment of *Candida* in the lungs of COVID-19 patients, including *Candida glabrata* (*Nakaseomyces glabrata)*, *Candida parapsilosis,* and *Candida albicans*. *Candida albicans* was the most commonly isolated fungi from the lungs through clinical microbiological culture. The enrichment of pathogenic *Candida* species we observed could potentially exacerbate inflammation and secondary infections in the lungs of COVID-19 patients. Antifungal treatments may need to be explored as an avenue for improving outcomes in severe COVID-19 cases with fungal co-infections.

In this study, we also investigated the potential factors driving microbiome variations. SARS-CoV-2 infection and clinical status accounted for both bacterial and fungal shifts in our multivariate analysis. Other associated clinical practices, such as oxygen support and clinical medication, may also contribute to the observed variations in univariate analysis. As antibiotic therapy was administered to all patients in our population, we did not find a specific association between bacterial microbiome and concurrent antibiotic therapy. However, antibiotic therapy influenced fungal composition, while antifungal treatment influenced the composition of both bacteria and fungi, indicating that bacteria–fungi interactions may play a role in the microbiome dysbiosis of COVID-19. Multiple inflammation indicators, known as rapid biomarkers for infections, have been suggested to correlate with the severity of the symptoms and clinical outcome of COVID-19 [45,46,47]. Many bacterial taxa enriched in COVID-19 showed significantly strong positive correlations with inflammation indicators, such as SAA, NWR, NLR, WBC, and NEUT. Collectively, these data suggest that co-infection with other pathogens, especially *Acinetobacter* genera, potentially complicates SARS-CoV-2 infection and leads to a worse outcome.

The inability to obtain data from healthy donors resulted in a lack of comparison between the COVID-19 patients and the healthy controls in our study. In a healthy human, the lung microbiota has a low density but harbors a prominent diversity of interacting microbiota. At the genus level, *Prevotella*, *Veillonella,* and *Streptococcus* are the predominant bacterial microorganisms, while the most common species of fungi found in lung tissue include *Cladosporium*, *Eurotium*, and *Aspergillus* [8,48]. Our study found that COVID-19 patients exhibited highly abundant bacteria, including *Acinetobacter*, *Klebsiella*, *Pseudomonas*, *Stenotrophomonas*, and *Escherichia*. Additionally, the most prevalent fungi were *Candida* and *Saccharomyces* in the lungs of COVID-19 patients. These results indicated a markedly different lung microbiota in COVID-19 patients and healthy individuals, as reported in previous studies [17,28].

In summary, we have mapped and characterized the lung microbiota of COVID-19 patients using rapid nanopore sequencing. We found decreased diversity and enrichment of opportunistic pathogens, especially *Acinetobacter baumannii* and *Candida* spp., in patients infected with SARS-CoV-2 compared to those with other common pneumonia. The enrichment of these pathogens suggests SARS-CoV-2 disrupts immune homeostasis in the lung, enabling blooms of fungi and bacteria that may in turn exacerbate damaging inflammation. Further research should delineate mechanisms by which the lung microbiome interacts with viral infection and inflammation to impact COVID-19 outcomes. Characterizing the lung microbial landscape via rapid nanopore sequencing represents a useful approach to identify potential prognostic indicators and therapeutic targets in this patient population.

## 5. Conclusions

Recent studies suggest important mutual interactions between viruses and the microbiota. However, the communication between lung microbiota and SARS-CoV-2, as well as the role of this association in diagnosis and treatment, are still unclear. In this study, we investigated the profiling of the bacterial and fungal lung microbiome in COVID-19 patients. COVID-19 patients exhibited differences in the biodiversity and composition of the lung microbiota compared to non-COVID-19 patients, characterized by the enrichment of opportunistic pathogens, particularly *Acinetobacter baumannii*. Analyzing the changes in the lung microbiota during SARS-CoV-2 infection may aid in the prediction of co-pathogens and contribute to the diagnosis and optimal treatment of SARS-CoV-2 respiratory infection during the current pandemic.

## Figures and Tables

**Table 1:** Clinical features of COVID-19 and non-COVID-19 patients.

	COVID-19 Cases (*n* = 38)	Non-COVID-19 Cases (*n* = 26)	*p*-Value
Sex [no. (%)]			
Female	9 (23.7)	7 (26.9)	1.000
Male	21 (76.3)	19 (73.1)	
Age, y			
Mean (Range)	61.2 (31–95)	57.0 (24–89)	0.360
Chronic medical illness [no. (%)]	29 (76.3)	20 (76.9)	1.000
Hypertension	9 (23.7)	10 (38.5)	0.321
Diabetes	8 (21.1)	5 (19.2)	1.000
Hyperlipidemia	2 (5.3)	0 (0.0)	0.648
Coronary artery disease	12 (31.6)	2 (7.7)	0.050
Chronic lung disease	0 (0.0)	2 (7.7)	0.315
Cancer	3 (7.9)	4 (15.4)	0.593
Pulmonary tuberculosis	5 (13.2)	2 (7.7)	0.779
HIV infection	0 (0.0)	3 (11.5)	0.123
HBV infection	1 (2.6)	2 (7.7)	0.735
Treatment [no. (%)]			
Oxygen support	36 (94.7)	14 (53.8)	0.001
Noninvasive ventilation	25 (65.8)	7 (26.9)	0.005
Invasive ventilation	35 (92.1)	12 (46.2)	0.001
ECMO	13 (34.2)	1 (3.8)	0.010
Antibiotic treatment	38 (100.0)	25 (100)	0.847
Antiviral treatment	26 (68.4)	3 (11.5)	0.001
Antifungal treatment	30 (78.9)	10 (38.5)	0.003
Clinical outcome [no. (%)]			
Alive	26 (68.4)	20 (76.9)	0.646
Died	12 (31.6)	6 (23.1)	
Clinical indicators [mean ± SD]			
WBC, ×10^9^/L	13.36 ± 8.51	10.30 ± 6.99	0.134
Neutrophil count, ×10^9^/L	12.13 ± 8.18	8.41 ± 6.73	0.060
Lymphocyte count, ×10^9^/L	0.56 ± 0.33	0.98 ± 0.69	0.002
Neutrophil/white blood cell ratio (%)	88.19 ± 9.88	76.63 ± 16.37	0.001
Neutrophil/lymphocyte ratio (%)	30.41 ± 28.03	18.96 ± 26.06	0.104
Hemoglobin level, g/L	97.31 ± 22.09	112.32 ± 24.77	0.015
Platelet count, ×10^9^/L	161.95 ± 104.03	183.27 ± 87.72	0.395
Alanine aminotransferase, U/L	58.61 ± 94.12	30.71 ± 25.83	0.194
Aspartate aminotransferase, U/L	56.89 ± 73.39	43.62 ± 42.94	0.464
Lactate dehydrogenase, U/L	475.00 ± 256.24	368.00 ± 320.32	0.395
Creatinine, μmol/L	76.11 ± 38.18	92.61 ± 37.55	0.127
D-dimer, mg/L	3698.00 ± 7220.31	2534.86 ± 4562.80	0.525
PCT, μg/L	1.47 ± 4.52	3.31 ± 6.35	0.191
CRP, mg/L	124.09 ± 148.84	54.65 ± 82.24	0.161
SAA, mg/L	134.31 ± 70.31	71.65 ± 72.69	0.048
IL-6, pg/mL	435.90 ± 1134.76	187.43 ± 303.47	0.358

Abbreviations: WBC, White blood cell count; PCT, procalcitonin; CRP, C-reactive protein; SAA, serum amyloid A; IL-6, interleukin-6.

## Data Availability

Metagenome sequences have been deposited in the Sequence Read Archive under Bioproject reference PRJNA720304: https://dataview.ncbi.nlm.nih.gov/object/PRJNA720304.

## Supplementary Materials

The following supporting information can be downloaded at: https://www.mdpi.com/article/10.3390/pathogens12070944/s1. Figure S1. Overview of the composition of the bacterial and fungal microbiome. Relative abundance at the genus level assigned to different bacterial (A) and fungal (B) taxa; Table S1. Detailed clinical information of 64 subjects in this study; Table S2. List of 64 clinical specimens and accession numbers of sequence data used in this study; Table S3. Organisms identified using clinical microbiological culture.
