# Supplementary figures and images for: Analysis of Lung Microbiome in COVID-19 Patients during Time of Hospitalization
Source: Pathogens. 2023 Jul 17;12(7):944. doi: 10.3390/pathogens12070944 (PMC10386632; doi:10.3390/pathogens12070944)

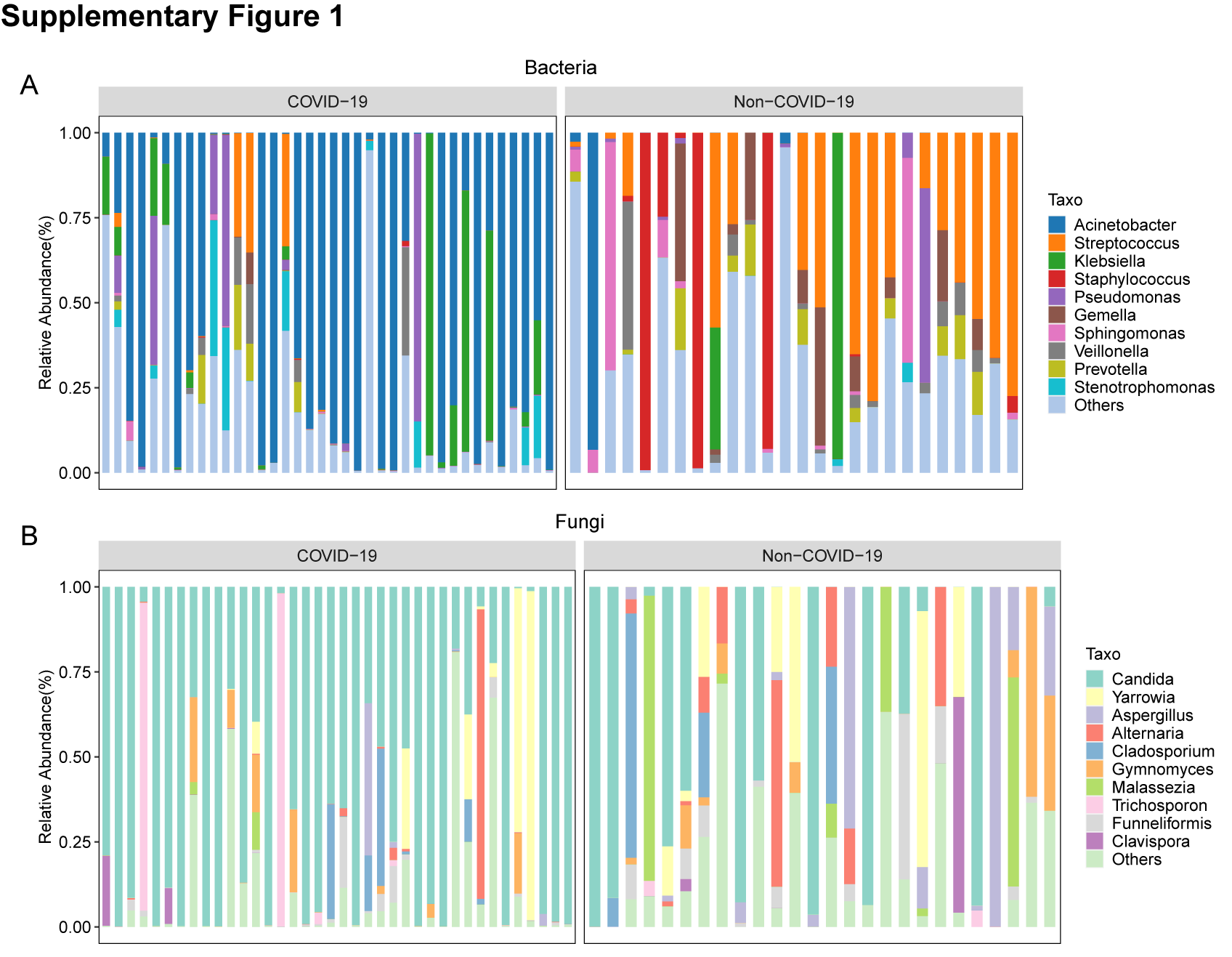

Supplement: Supplementary file 1 [file pathogens-12-00944-s001.zip › Supplementary Figure S1.tif]
